# Plant Peroxisomes: A Factory of Reactive Species

**DOI:** 10.3389/fpls.2020.00853

**Published:** 2020-07-03

**Authors:** Francisco J. Corpas, Salvador González-Gordo, José M. Palma

**Affiliations:** Group of Antioxidants, Free Radicals and Nitric Oxide in Biotechnology, Food and Agriculture, Department of Biochemistry, Cell and Molecular Biology of Plants, Estación Experimental del Zaidín, Consejo Superior de Investigaciones Científicas (CSIC), Granada, Spain

**Keywords:** catalase, reactive oxygen, nitrogen and sulfur species, superoxide dismutase, nitric oxide, *S*-nitrosation, persulfidation

## Abstract

Plant peroxisomes are organelles enclosed by a single membrane whose biochemical composition has the capacity to adapt depending on the plant tissue, developmental stage, as well as internal and external cellular stimuli. Apart from the peroxisomal metabolism of reactive oxygen species (ROS), discovered several decades ago, new molecules with signaling potential, including nitric oxide (NO) and hydrogen sulfide (H_2_S), have been detected in these organelles in recent years. These molecules generate a family of derived molecules, called reactive nitrogen species (RNS) and reactive sulfur species (RSS), whose peroxisomal metabolism is autoregulated through posttranslational modifications (PTMs) such as *S*-nitrosation, nitration and persulfidation. The peroxisomal metabolism of these reactive species, which can be weaponized against pathogens, is susceptible to modification in response to external stimuli. This review aims to provide up-to-date information on crosstalk between these reactive species families and peroxisomes, as well as on their cellular environment in light of the well-recognized signaling properties of H_2_O_2_, NO and H_2_S.

## Introduction

For many years, peroxisomes in higher plants have been given different names, such as glyoxysomes during seed germination and leaf senescence, as well as leaf, root and fruit peroxisomes according to their presence in different organs and at different physiological stages (Tolbert and Essner, 1981; Palma et al., 2018). This is explained by the presence of metabolic pathways which appear to be specific to each type of peroxisome. However, peroxisomes, which share a number of metabolites and enzymes common to all types of peroxisome, is now the preferred term regardless of their specific metabolic characteristics (Pracharoenwattana and Smith, 2008). The most noteworthy metabolites and enzymes include H_2_O_2_ and catalase, which are directly involved in the metabolism of reactive oxygen species (ROS) (Su et al., 2018; Sousa et al., 2019).

Peroxisomes have a simple morphological constitution composed of a single membrane surrounding an amorphous matrix. Over the last 30 years, an increasing number of new and often unexpected components and processes in these organelles have been identified (Bueno and del Río, 1992; del Río et al., 1992; Corpas et al., 1994, 2001, 2017a, 2019a, Barroso et al., 1999; Reumann et al., 2009; Clastre et al., 2011; Simkin et al., 2011; Chowdhary et al., 2012; Guirimand et al., 2012; Oikawa et al., 2015; Reumann and Bartel, 2016; Kao et al., 2018; Pan et al., 2018, 2020; Borek et al., 2019), indicating that the plant peroxisomal metabolism and consequently peroxisomal enzymatic and non-enzymatic components are more diverse than previously predicted. The diverse complementary range of experimental approaches used to identify these new peroxisomal constituents includes: (i) the biochemical, proteomic and molecular analysis of purified peroxisomes combined with bioinformatics methodologies and (ii) cell biology studies of features such as immune localization with the aid of electron microscopy and specific fluorescent probes with appropriated controls. Although the model plant *Arabidopsis thaliana* has increased our knowledge of plant peroxisomes, it should be pointed out that studies of peroxisomes from other plant species have been essential, as the peroxisomal metabolism can be modulated depending on the plant organ, development time and plant species involved. Therefore, this review principally aims to provide an update of research on the metabolism of reactive species associated with oxygen, nitrogen and, more recently, sulfur, as well as to outline new challenges and possible future research perspectives regarding crosstalk between peroxisomes and other subcellular compartments such as oil bodies, mitochondria and plastids which are closely related both biochemically and structurally (Palma et al., 2006; Oikawa et al., 2019). Information on plant peroxisomes could also be useful in relation to peroxisome research into other organisms and vice versa.

## Peroxisomal ROS Metabolism

**FIGURE 1:**
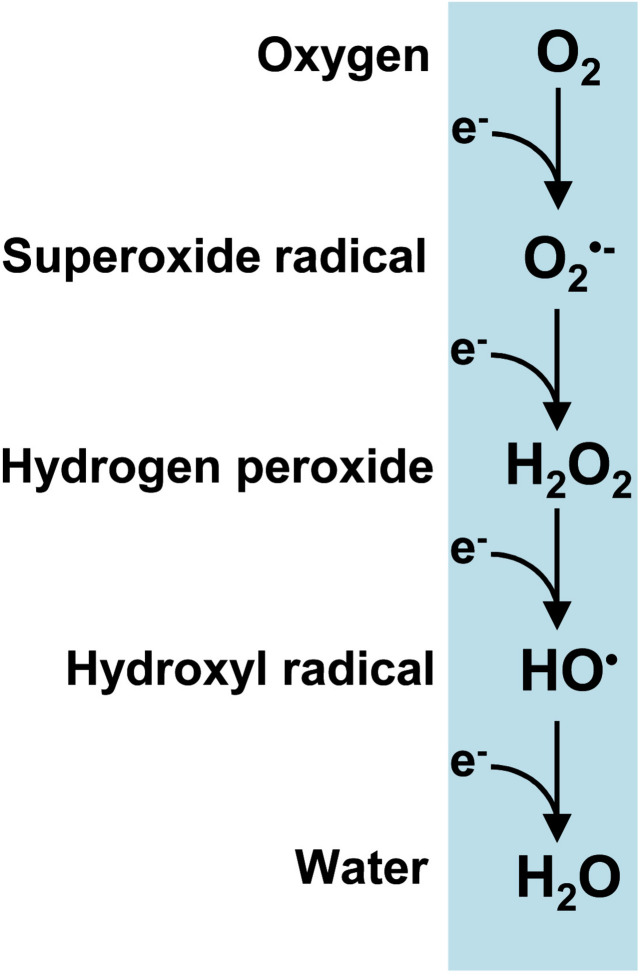
Reactive oxygen species (ROS) produced from a sequential one-electron reduction from oxygen.

Reactive oxygen species (ROS) are produced by a series of single-electron reductions in molecular oxygen which sequentially form superoxide (O_2_^•–^), hydrogen peroxide (H_2_O_2_) and hydroxyl (HO^•^) radicals and ultimately ending in water (Figure 1). It is worth noting that the term peroxisomes, formerly known as microbodies, originates from their high H_2_O_2_ content (De Duve and Baudhuin, 1966; Corpas, 2015). Plant peroxisomes contain a significant number of enzymatic systems capable of generating H_2_O_2_ such as glycolate oxidase (GOX), acyl-CoA oxidase (AOX), urate oxidase (UO), polyamine oxidase, copper amine oxidase (CuAO), sulfite oxidase (SO), sarcosine oxidase (SOX), or superoxide dismutase (SOD) (Hauck et al., 2014; Corpas et al., 2017a and references therein). These H_2_O_2_-generating enzymes are involved in multiple biochemical pathways which are essential not only for the endogenous metabolism of plant peroxisomes but also for their interactions with other subcellular compartments such as plastids, mitochondria, cytosols, oil bodies and nuclei. In these subcellular interconnections, H_2_O_2_ itself plays a highly important role as a signal molecule in crosstalk between organelles in order to coordinate cell function.

Photorespiration has been estimated to be responsible for 70% of total H_2_O_2_ generated mainly from peroxisomal GOX in photosynthetic tissues (Noctor et al., 2002). Zhang et al. (2016) have described an elegant dynamic physical GOX-catalase association-dissociation mechanism that fine-tunes peroxisomal H_2_O_2_ in rice plants. Although peroxisomal H_2_O_2_ is kept under control when GOX and catalase are associated, under stress conditions and when mediated by salicylic acid (SA), this complex GOX-catalase dissociation mechanism inhibits catalase activity, leading to an increase in cellular H_2_O_2_ which acts as a signaling molecule (Zhang et al., 2016; Kohli et al., 2019). Another sophisticated mechanism, involving the interaction of the γb protein from the barley stripe mosaic virus with GOX, has been reported to inhibit GOX and to facilitate infection with the virus (Yang et al., 2018). More recently, Yamauchi et al. (2019) observed a connection between the H_2_O_2_-generating GOX and catalase, which is required in the stomatal movement. Thus, when there is an increase of oxidized peroxisomes they were removed by pexophagy allowing an increase in H_2_O_2_ in guard cells which mediated the stomatal closure. This mechanism of ROS homeostasis in guard cells seems to be relevant in response to environmental changes. On the other hand, the new peroxisomal small heat shock protein Hsp17.6CII, capable of increasing catalase activity especially under stress conditions, has been reported to be present in Arabidopsis plants (Li et al., 2017).

Acyl-CoA oxidase is another key peroxisomal H_2_O_2_-generating enzyme involved in fatty acid β-oxidation which, in collaboration with lipid bodies, enables triacylglyceride mobilization especially during seed germination and is also involved in the synthesis of signal molecules such as jasmonic acid (Baker et al., 2006; Chen et al., 2019b; Wang X. et al., 2019; Xin et al., 2019). However, under stress conditions such as salinity, ROS generated by peroxisomal fatty acid β-oxidation have a negative impact and contribute to oxidative damage (Yu et al., 2019).

Polyamines such as putrescine, spermidine and spermine are well known to be involved in multiple physiological processes, as well as mechanisms of response to various stress conditions (Wuddineh et al., 2018; Chen et al., 2019a; Wang W. et al., 2019). Several enzymes involved in the catabolism of polyamine, including H_2_O_2_-producing polyamine oxidase (PAO) and copper amino oxidase (CuAO), have been reported to be present in plant peroxisomes (Moschou et al., 2008; Kusano et al., 2015). These enzymes are also involved in the γ-aminobutyric acid (GABA) biosynthesis signaling pathway (Zarei et al., 2015; Corpas et al., 2009b).

In addition, peroxisomal xanthine oxidoreductase (XOR) and superoxide dismutase (SOD), key enzymes in O_2_^•–^ and H_2_O_2_ metabolism, can be regulated by stress conditions such as salinity, heavy metal and ozone stress (Corpas et al., 1993, 2008; Ueda et al., 2013).

Although catalase is the principal antioxidant enzyme in the matrix of all types of peroxisome (Mhamdi et al., 2010, 2012; Palma et al., 2020 and references therein), other enzymatic antioxidants are present in both the matrix and the membrane. It is also important to highlight the role of SOD isozymes, which differ according to peroxisomal origin (del Río et al., 2018). Thus, peroxisomes of watermelon cotyledons have two SOD isoenzymes, a CuZn-SOD located in the matrix and a Mn-SOD that is bound to the membrane (Bueno and del Río, 1992; Rodríguez-Serrano et al., 2007); pea leaf peroxisomes have a Mn-SOD present in the matrix; sunflower cotyledon peroxisomes have only a CuZn-SOD which is also located in the matrix (Corpas et al., 1998); carnation petal and pepper fruit peroxisomes have a Mn- and an Fe-SOD (Droillard and Paulin, 1990; Palma et al., 2018); and olive fruits peroxisomes contain four SOD isozymes, an Fe-SOD, two CuZn-SOD and a Mn-SOD (López-Huertas and del Río, 2014). Therefore, it could be hypothesized that the presence of two or more types of SOD in peroxisomes must have some physiological advantages. Thus, one of the SOD isozymes could be constitutive while the other one could be inducible under environmental or physiological stimuli such as seedling development, leaf senescence or fruit ripening.

**FIGURE 2:**
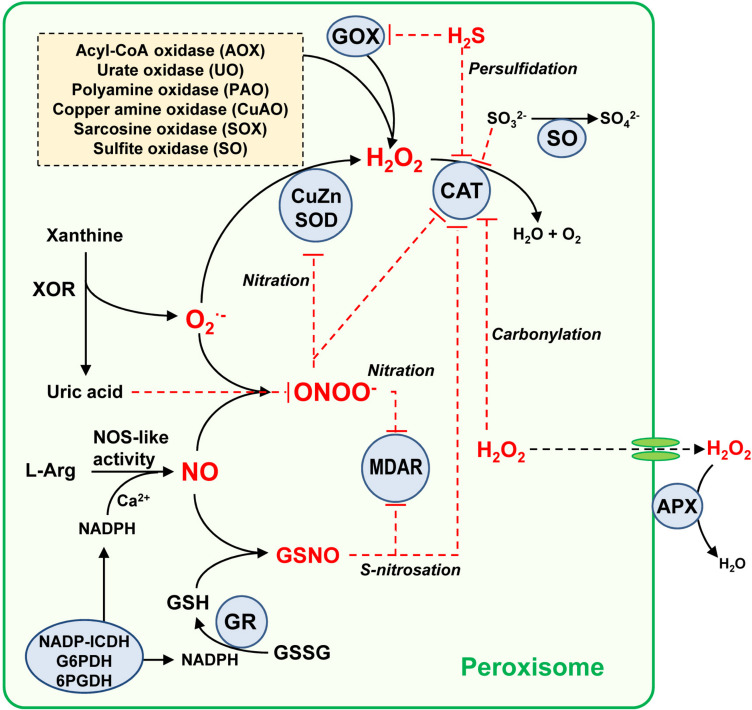
Simple model of the global metabolism of reactive oxygen/nitrogen/sulfur species in plant peroxisomes. Peroxisomes have an important battery of H_2_O_2_-generating enzymes, being the photorespiratory glycolate oxidase (GOX) one of the most relevant. Peroxisomal xanthine oxidoreductase (XOR) activity generates uric acid which the concomitant generation of superoxide radical (O_2_^•–^) which is dismutated to H_2_O_2_ by superoxide dismutase (SOD). All three SOD types have been described in plant peroxisomes from different origin, CuZ-SOD, Mn-SOD, and Fe-SOD. The H_2_O_2_ pool is mainly decomposed by catalase (CAT) but also by the membrane-bound ascorbate peroxidase (APX). An L-arginine (L-Arg) and Ca^2+^ dependent NOS-like activity generates NO which can react chemically with O_2_^–^ to produce peroxynitrite (ONOO^–^), a nitrating molecule that facilitates PTMs such as tyrosine nitration. NO can also interact with reduced glutathione (GSH) to form *S*-nitrosoglutathione (GSNO), a NO donor which mediates *S*-nitrosation. GSH is regenerated by glutathione reductase (GR) which requires NADPH supplied by several NADPH-generating enzymes (NADPH-ICDH, G6PDH, and 6PGDH). Uric acid is a ONOO^–^ scavenger, this being a mechanism of peroxisomal auto-regulation. With all these components, and according to reported data, the peroxisomal targets of NO-derived PTMs identified so far are CAT, CuZn-SOD, and monodehydroascorbate reductase (MDAR) which can undergo an inhibitory effect either by nitration or *S*-nitrosation. Additionally, CAT and GOX can be inhibited by hydrogen sulfide (H_2_S), and CAT is also inhibited by carbonylation. The H_2_O_2_-generating sulfite oxidase (SO) converts sulfite (SO_3_^2–^) to sulfate (SO_4_^2–^), which is a mechanism of protection because sulfite inhibits catalase activity. Red line denotes inhibition effect.

In addition, it is worth noting the role of ascorbate-glutathione cycle components, including ascorbate peroxidase (APX), monodehydroascorbate reductase (MDAR), dehydroasrcorbate reductase (DAR) and glutathione reductase (GR) (Jiménez et al., 1998; Romero-Puertas et al., 2006; López-Huertas and del Río, 2014; Corpas et al., 2017a). While MDAR is present in both matrix and membrane (Leterrier et al., 2005; Lisenbee et al., 2005; Eastmond, 2007), APX is exclusively located in the membrane (Corpas et al., 1994; Yamaguchi et al., 1995; Bunkelmann and Trelease, 1996). With its high affinity for H_2_O_2_ (low *Km* value around 74 μM), membrane-bound APX appears to have fine-tuned control of H_2_O_2_ (Ishikawa et al., 1998) as compared to catalase, which, with a *Km* value in the mM range, is less efficient at low concentrations of H_2_O_2_ (Huang et al., 1983; Mhamdi et al., 2010). The *Km* values for plant catalase are reported to vary quite considerably, with, for example, a *Km* of 50 mM in *Beta vulgaris* (Dinçer and Aydemir, 2001), 100 mM in rice (Ray et al., 2012) and 190 mM in pea (del Río et al., 1977). Peroxisomal APX appears to be critical in a diverse range of processes such as seedling development (Corpas and Trelease, 1998) and leaf senescence (Ribeiro et al., 2017). To maintain the ascorbate-glutathione cycle at the GR level, NADPH needs to be supplied by NADP-dependent endogenous dehydrogenases including glucose-6-phosphate dehydrogenase (G6PDH), 6-phosphogluconate dehydrogenase (6PGDH) and isocitrate dehydrogenase (NADP-ICDH) (Leterrier et al., 2016; Corpas and Barroso, 2018b and references therein). In addition, Corpas et al. (2017b) have reported the presence of a protein immunologically related to plant peroxiredoxins, whose expression is differentially modulated under oxidative stresses such as those induced by CdCl_2_ and the herbicide 2,4-dichlorophenoxyacetic acid (2,4-D); however, further research is necessary to clarify this phenomenon. Figure 2 shows a working model of the ROS metabolism and its interaction with other reactive species, including NO and H_2_S, which modulate the activity of peroxisomal enzymes through posttranslational modifications (PTMs), events which will be further discussed below.

Given the capacity of ROS to mediate several PTMs, particularly carbonylation and *S*-sulfenylation, certain amino acid residues, especially arginine, lysine, threonine and proline, are carbonylated, which affects target protein function in many cases (Debska et al., 2012; Lounifi et al., 2013). Several studies have identified peroxisomal proteins, such as catalase, malate synthase and the fatty acid β-oxidation multifunctional protein AIM1, which undergo carbonylation (Nguyen and Donaldson, 2005; Anand et al., 2009; Mano et al., 2014; Rodríguez-Ruiz et al., 2019). On the other hand, H_2_O_2_ can oxidize specific protein cysteine thiols to sulfenic acid (SOH), a process known as *S*-sulfenylation, which usually results in enzymatic inactivation. Using proteomic techniques, approximately 2% of peroxisomal proteins have been reported to be susceptible to *S*-sulfenylation (Akter et al., 2017; Huang et al., 2019). This PTM has been observed to occur with respect to fatty acid β-oxidation acyl-coenzyme A oxidase 1, the multifunctional proteins MFP2, and AIM1, as well as amine oxidase, phosphomevalonate kinase, MDAR and NADP-ICDH. Table 1 shows a summary of peroxisomal enzymes targeted by carbonylation and *S*-sulfenylation, as well as other PTMs mediated by RNS and RSS, a subject which will be discussed below.

**TABLE 1:** Peroxisomal enzymes target of diverse posttranslational modifications (PTMs) whose activities are affected by either ROS, RNS, or RSS.

Peroxisomal enzyme	Pathway/Reaction	PTM	Effect on activity
Catalase (CAT)	H_2_O_2_ decomposition	Carbonylation	Inhibition
		Tyr-nitration	Inhibition
		*S*-nitrosation	Inhibition
		Persufidation	Inhibition
Monodehydroascorbate reductase (MDAR)	Ascorbate-glutathione cycle	Tyr-nitration	Inhibition
		*S*-nitrosation	Inhibition
		*S*-sulfenylation^a^	Not reported
Hydroxypyruvate reductase (HPR)	Photorespiration	Tyr-nitration	Inhibition
		*S*-nitrosation	Inhibition
Glycolate oxidase (GOX)	Photorespiration	*S*-nitrosation	Inhibition
		Persufidation	Inhibition
CuZn-superoxide dismutase (CSD3)	O_2_^•–^ dismutation	Tyr-nitration	Inhibition
Malate dehydrogenase (MDH)	Fatty acid β-oxidation	Tyr-nitration	Inhibition
		*S*-nitrosation	Inhibition
Malate synthase (MS)	Glyoxylate cycle	Carbonylation	Inhibition
Isocitrate lyase (ICL)	Glyoxylate cycle	S-nitrosation^a^	Not reported
Acyl-coenzyme A oxidase 1	Fatty acid β-oxidation	Persulfidation^a^	Not reported
		*S*-sulfenylation^a^	Not reported
Multifunctional protein AIM1 isoform	Fatty acid β-oxidation	S-nitrosation^a^	Not reported
		*S*-sulfenylation^a^	Not reported
Lon protease homolog 2	Peroxisomal protein import	*S*-nitrosation^a^	Not reported
Phosphomevalonate kinase	Isoprenoid biosynthesis	*S*-sulfenylation^a^	Not reported
NADP-isocitrate dehydrogenase	NADPH supply	Tyr-nitration	Inhibition
		*S*-nitrosation	Inhibition
		Persufidation	Inhibition
		*S*-sulfenylation^a^	Not reported

*^a^Proteomic identification.*

Given growing awareness of the important role of ROS peroxisomal metabolism in combating biotic stress, the expression of genes encoding for peroxisomal proteins involved in their biogenesis, fatty acid catabolism and the H_2_O_2_-generating glyoxylate cycle have been reported to increase during interactions between the pathogen *Sclerotinia sclerotiorum* and rapeseed (*Brassica napus*), thus facilitating pathogen cell wall degradation and metabolism detoxification (Chittem et al., 2020). On the other hand, using the Arabidopsis *nca1* mutant with no catalase activity 1, containing residual activity of the three catalase isozymes, Hackenberg et al. (2013) identified a link between catalase and ROS production as autophagy-dependent cell death progresses. Table 2 shows some functional implications of peroxisomal H_2_O_2_ and other signal molecules generated in this organelle.

**TABLE 2:** Signal molecules generated in plant peroxisomes during different processes and their functional implications.

Peroxisomal signal	Functional implication	References
Hydrogen peroxide (H_2_O_2_)	Plant development and stress response	Zhang et al., 2016; Su et al., 2019
	Involved in peroxisome abundance under drought and heat stress	Hinojosa et al., 2019
	Pexophagy	Yamauchi et al., 2019
	Pathogen defense	Chittem et al., 2020
Nitric oxide (NO)	Pollen tube development	Prado et al., 2004
	Leaf senescence	Corpas et al., 2019
	Lateral root formation	Schlicht et al., 2013
	Heavy metal and root architecture	Piacentini et al., 2020
Hydrogen sulfide	Regulation of catalase	Corpas et al., 2019a
(H_2_S)	Herbicide glyphosate response	
Jasmonic acid (JA)	Plant growth	Wang X. et al., 2019
	Environmental stimuli	Xin et al., 2019
	Insect defense	
γ-aminobutyric acid (GABA)	Fruit flavor and flower fragrance	Zarei et al., 2015
	Abiotic stress tolerance	Shelp and Zarei, 2017

The generation of singlet oxygen (^1^O_2_) has always been associated with chloroplasts, particularly in photosystem II, responsible for various types of photo-damage which triggers distinct cellular responses (Wagner et al., 2004; Rosenwasser et al., 2011; Chen and Fluhr, 2018; Dogra et al., 2018). Using the green fluorescence probe to detect ^1^O_2_, peroxisomes, mitochondria and nuclei have been shown to be either the origin or target of ^1^O_2_, suggesting that this ROS is generated in a light-independent manner (Mor et al., 2014). These findings open up new questions about the importance of ^1^O_2_ in the mechanism of response to plant stress in which several subcellular compartments including peroxisomes are involved.

## Peroxisomal Reactive Nitrogen Species (RNS)

**FIGURE 3:**
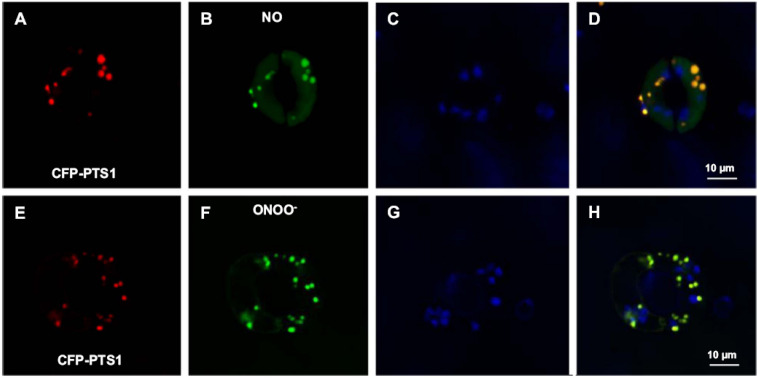
Representative images illustrating the CLSM *in vivo* detection of nitric oxide (NO) and peroxynitrite (ONOO^–^), peroxisomes (red) and chloroplasts (blue) in guard cells of transgenic Arabidopsis seedlings expressing CFP-PTS1. **(A,E)** Fluorescence punctate (red) attributable to CFP-PTS1, indicating the localization of peroxisomes in guard cells. **(B,F)** Fluorescence punctate (green) attributable to the detection in the same guard cells of NO and ONOO^–^, respectively. **(C,G)** Chlorophyll autofluorescence (blue) attributable to the detection of chloroplasts. **(D,H)** Merged images for corresponding panels. Reproduced with permission from Corpas et al. (2017a) provided by Elsevier.

Nitric oxide (NO) metabolism has a significant impact on cellular metabolisms due to its involvement in the important plant physiological processes of seed and pollen germination, root development, stomatal closure, senescence and fruit ripening, as well as in the mechanism of response to many environmental stresses including salinity, drought, heavy metals and extreme temperature (Neill et al., 2008; León et al., 2014; Begara-Morales et al., 2018; Kolbert et al., 2019; Wei et al., 2020). NO belongs to a family of related molecules called reactive nitrogen species (RNS), with peroxynitrite (ONOO^–^) and *S*-nitrosogluthione (GSNO) being the most studied. Using various experimental approaches including electron paramagnetic resonance (EPR) spectroscopy, as well as biochemical and cellular biology, some RNS including NO, ONOO^–^ and GSNO have been detected in plant peroxisomes (Barroso et al., 2013; Corpas and Barroso, 2014b; Corpas et al., 2019). Identification of peroxisomal proteins undergoing PTMs mediated by these NO-derived species is strong evidence of an active RNS metabolism in peroxisomes. Figures 3A–H shows *in vivo* images of NO and ONOO^–^ in Arabidopsis guard cell peroxisomes detected by confocal laser scanning microscopy (CLSM) and specific fluorescent probes.

ONOO^–^ results from a reaction between NO with O_2_^•–^, considered one of the fastest chemical reactions with a rate constant (k) of 1.9 × 10^10^ M^–1^ s^–1^ (Kissner et al., 1997). ONOO^–^, a strong oxidant and nitrating molecule involved in protein tyrosine nitration (NO_2_-Tyr), modifies protein function, mostly through inhibition (Corpas et al., 2009a; Mata-Pérez et al., 2016). This NO-derived PTM involves the covalent oxidative addition of a nitro group (-NO_2_) to tyrosine residues, a highly selective process which depends on factors such as the protein environment of the Tyr and the nitration mechanism (Bartesaghi and Radi, 2018). Table 1 shows some nitrated proteins identified in plant peroxisomes and how their function is affected. Interestingly, some of the proteins affected are directly involved in the ROS metabolism, indicating a close metabolic interconnection between both families of reactive species.

The antioxidant glutathione (GSH), a tripeptide (γ-Glu-Cys-Gly), undergoes *S*-nitrosation in order to generate GSNO, a low-molecular-weight NO reservoir, through a covalent addition of NO to the thiol group of Cys residues in order to form *S*-nitrosothiol (SNO) (Airaki et al., 2011). GSNO is a key molecule given its dynamic interaction with free cysteines, GSH and proteins through processes such as *S*-nitrosation, *S*-transnitrosation and *S*-glutathionylation (Broniowska et al., 2013; Corpas et al., 2013a, b). GSNO is enzymatically decomposed by GSNO reductase (GSNOR; Leterrier et al., 2011), an enzyme susceptible to *S*-nitrosation and consequently inhibition (Guerra et al., 2016). An increase in Tyr nitration, an irreversible process, is usually associated with nitro-oxidative stress; however, protein *S*-nitrosation, a reversible process, is a regulatory protein mechanism that occurs under physiological and stress conditions. Table 1 shows some peroxisomal proteins targeted by *S*-nitrosation, as well as proteins involved in ROS metabolism which are targeted by these NO-mediated PTMs.

The number of peroxisomal proteins targeted by NO-mediated PTMs is growing continuously. Using the biotin-switch technique and liquid chromatography/mass spectrometry/mass spectrometry (LC-MS/MS), several more *S*-nitrosated peroxisomal proteins have been identified during adventitious root growth induced by treatment with NO (Niu et al., 2019). These proteins include the peroxisomal LON2 protease, which is necessary for matrix protein import into peroxisomes (Lingard and Bartel, 2009); isocitrate lyase (ICL), involved in the glyoxylate cycle; and the multifunctional AIM1-like isoform, involved in fatty acid β-oxidation.

However, the source of enzymatic NO, as yet unelucidated, is currently the most controversial aspect of NO metabolism in higher plants (Kolbert et al., 2019). Two main candidates have been proposed: nitrate reductase (NR) (Mohn et al., 2019) and L-arginine-dependent NO synthase-like activity (Corpas et al., 2017a). Although no evidence of NR has been found in plant peroxisomes, NO synthase-like activity has been found and characterized in peroxisomes purified from pea leaves (Barroso et al., 1999). Though as yet unidentified, this protein is called NOS-like activity, as peroxisomal NO generation requires NOS proteins similar to those found in animals, including L-arginine, NADPH, FMN, FAD, tetrahydrobiopterin, calcium, and calmodulin (Corpas and Barroso, 2017b; Corpas et al., 2019). The protein responsible for NO generation is imported by a type 2 peroxisomal targeting signal involving a process dependent on calmodulin and calcium (Corpas and Barroso, 2014a, 2018a).

Peroxisomal NO metabolism is involved in processes such as pollen tube germination (Prado et al., 2004), lateral root formation (Schlicht et al., 2013), and leaf senescence (Corpas et al., 2019), as well as in responses to environmental and heavy metal stresses such as salinity (Corpas et al., 2009b), lead (Corpas and Barroso, 2017a), and cadmium (Corpas and Barroso, 2014b; Piacentini et al., 2020).

## Reactive Sulfur Species (RSS) in Plant Peroxisomes

Reactive sulfur species (RSS) are chemically comparable to ROS (Olson, 2019) and can be generated from hydrogen sulfide (H_2_S), some of these species are thiyl radical (HS^•^), hydrogen persulfide (H_2_S_2_), persulfide radical (HS_2_^•–^), sulfite (SO_3_^2–^) or sulfate (SO_4_^2–^) among others (Gruhlke and Slusarenko, 2012; Ono et al., 2014; Mishanina et al., 2015; Park et al., 2015; Schöneich, 2016). However, the biochemistry of H_2_S in cells, given its multiple interactions with other reactive species, is more complex than previously thought (see Filipovic et al., 2018 for a more in-depth review); for example, protein thiyl radicals are generated during the reaction of H_2_O_2_ with heme proteins, possibly inducing protein degradation (Schöneich, 2016).

Different molecules and enzymes, such as GSH (Müller et al., 2004), glutathione reductase (Romero-Puertas et al., 2006), and sulfite oxidase (Nowak et al., 2004; Hänsch and Mendel, 2005), involved in sulfur metabolism, are present in plant peroxisomes. Sulfite oxidase (SO) catalyzes the conversion of sulfite to sulfate by producing H_2_O_2_. The functional relevance of this enzyme is that it can protect catalase activity since sulfite, at low concentration, has the capacity to inhibit catalase activity (Veljovic-Jovanovic et al., 1998). Nevertheless, despite the greater importance attributed to peroxisomal SO in a recent study, mitochondrial SO in animal cells has the capacity to generate NO from nitrite (Bender et al., 2019), while NO enzymatic generation from SO in plant peroxisomes remains to be proven. An earlier study confirmed the important role played by the peroxisomal RSS metabolism (Corpas and Barroso, 2015).

**FIGURE 4:**
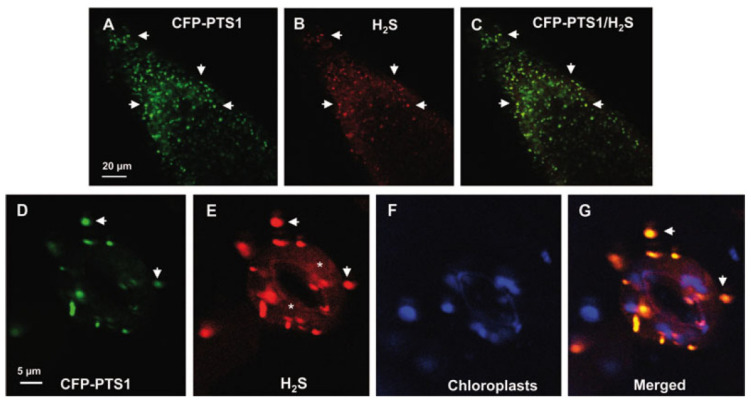
Representative images illustrating the CLSM *in vivo* detection of H_2_S (red color) and peroxisomes (green color) in root tips **(A–C)** and guard cells **(D–G)** of 10 days old Arabidopsis seedlings expressing CFP-PTS1. **(A,D)** Fluorescence puncta (green) attributable to CFP-PTS1, indicating the localization of peroxisomes. **(B,E)** Fluorescence punctate (red) attributable to H_2_S detection in the same area. **(C)** Merged image of **(A,B)** showing colocalized fluorescence punctate (yellow). **(F)** Chlorophyll autofluorescence (blue) demonstrating location of chloroplasts. **(G)** Merged images of **(D–F)**. H_2_S (red color) was detected by using 5 mM WSP-5, a fluorescence probe for H_2_S. Arrows indicate representative punctate spots corresponding to the colocalization of H_2_S with peroxisomes. Asterisks indicate localization of H_2_S in the cytosol. Reproduced with permission from Corpas et al. (2019a) provided by John Wiley and Sons.

H_2_S has recently been proven to be present in plant peroxisomes (Corpas et al., 2019a). Figures 4A–G shows representative images of H_2_S in peroxisomes from the root tips and guard cells of Arabidopsis seedlings detected by *in vivo* CLSM and a specific fluorescent probe. Using proteomic techniques, some peroxisomal enzymes have been identified as targets of persulfidation (Aroca et al., 2015, 2017). On the other hand, *in vitro* analysis shows that catalase activity from Arabidopsis and sweet pepper fruits is inhibited in the presence of H_2_S (Corpas et al., 2019a). Although, to our knowledge, the enzymatic source of peroxisomal H_2_S remains unknown, previous studies have proposed some potential candidates. For example, catalase, which functions as a sulfide oxidase or sulfur reductase, is capable of oxidizing or generating H_2_S (Olson et al., 2017). SOD has also been reported to have the capacity to catalyze the reaction between O_2_ and H_2_S to generate persulfide (Olson et al., 2018). In a previous study by Corpas and Barroso (2015), the presence of these enzymatic and non-enzymatic components in plant peroxisomes indicates that, in addition to ROS and RNS, these organelles also have an active RSS metabolism.

## Crosstalk Between Peroxisomal Reactive Species

Functional interactions and inter-regulation through PTMs in these families of reactive species are shown in Figure 2. In this working model, under physiological conditions, catalase, the main antioxidant enzyme, regulates levels of H_2_O_2_ generated by different pathways, principally photorespiratory glycolate oxidase (GOX) (Noctor et al., 2002). On the other hand, peroxisomal xanthine oxidoreductase (XOR) activity involved in purine catabolism generates uric acid, with the concomitant formation of the O_2_^•–^ (Corpas et al., 1997, 2008; Zarepour et al., 2010), which, in turn, is dismutated to H_2_O_2_ by SOD. The pool of H_2_O_2_ is mainly decomposed by catalase (CAT) and also by membrane-bound ascorbate peroxidase (APX). L-Arg-dependent NOS-like activity generates NO (Corpas et al., 2019) which chemically reacts with O_2_^•–^ to produce peroxynitrite (ONOO^–^), a nitrating molecule that facilitates PTMs such as tyrosine nitration. NO also interacts with reduced glutathione (GSH) to form *S*-nitrosoglutathione (GSNO), a NO donor that mediates *S*-nitrosation. Uric acid is a physiological ONOO^–^ scavenger (Alamillo and García-Olmedo, 2001) involved in endogenous peroxisomal auto-regulation. Thus, the peroxisomal enzymes targeted by NO-derived PTMs, catalase (CAT), CuZn-SOD, and monodehydroascorbate reductase (MDAR), are inhibited by nitration and S-nitrosation. Both CAT, and GOX are inhibited by H_2_S; the former is also inhibited by carbonylation when H_2_O_2_ is overproduced. In addition, H_2_O_2_-generating sulfite oxidase (SO) is involved in the conversion of sulfite to sulfate which, given sulfite’s ability to inhibit SO, has been reported to be a catalase protection mechanism. These interconnections highlight the biochemical complexity of this self-regulated plant peroxisome network, in which the antioxidant catalase is one of the most regulated peroxisomal enzymes (Palma et al., 2020).

## Conclusion

Much of our knowledge of reactive species metabolism in plant peroxisomes is now well established. The three molecular families ROS, RNS, and RSS are present in plant peroxisomes, which are considered to be potential producers of reactive species and to play an important role in the cell signaling network. However, our limited knowledge of reactive species families needs to be expanded by identifying new peroxisomal protein targets. We also need to determine the effect of the different PTMs, carbonylation, *S*-sulfenylation, *S*-nitrosation, tyrosine nitration, and persulfidation, on target protein function and peroxisomal metabolism. In addition, interactions with other subcellular compartments which share biochemical pathways such as photorespiration, fatty acid β-oxidation, isoprenoid biosynthesis and purine and polyamine metabolism (Clastre et al., 2011; Guirimand et al., 2012; Corpas et al., 2019b) should be investigated. Similarly, the relationship between reactive species and complex peroxisomal biogenesis, division and matrix/membrane protein import mechanisms (Reumann and Bartel, 2016; Kao et al., 2018) has been underexplored (López-Huertas et al., 2000). Further research should also be carried out to identify the proteins responsible for endogenous peroxisomal generation of NO and H_2_S. This would increase our knowledge of how organelle biochemistry is modulated within the framework of the whole cell metabolism. This research could lead to biotechnological applications given the important role of peroxisomes in many physiological processes and in responses to biotic and abiotic stresses. Furthermore, in addition to harboring reactive species, with their known signaling properties, peroxisomes are a source of other signaling molecules such as jasmonic acid and γ-aminibutic acid (GABA), which extends the functional role of plant peroxisomes. Table 2 shows signaling molecules generated in the plant peroxisomal metabolism and some examples of their role in various plant processes.

## Author Contributions

Authors have made a collaborative, direct and intellectual contribution to the work, and have approved it for publication.

## Conflict of Interest

The authors declare that the research was conducted in the absence of any commercial or financial relationships that could be construed as a potential conflict of interest.
